# Mucormycosis Necrotizing Soft Tissue Infection: A Case Report of Fungal Infection Following a High-Speed Motorcycle Accident

**DOI:** 10.7759/cureus.35896

**Published:** 2023-03-08

**Authors:** Justin Taylor, Steven Vuu, Darwin Ang

**Affiliations:** 1 Medical School, University of Central Florida College of Medicine, Orlando, USA; 2 General Surgery, University of Central Florida College of Medicine, Orlando, USA; 3 Trauma, HCA Healthcare, Ocala, USA; 4 Trauma, University of South Florida, Tampa, USA

**Keywords:** debridement, fungal infection, polytrauma, necrotizing fasciitis, motorcycle accident, cutaneous mucormycosis

## Abstract

Mucormycosis is a rare fungal infection characterized by rapidly progressing infarction and necrosis of host tissue, frequently resulting in death. It is most well-known for causing a devastating rhinocerebral infection, however, cutaneous mucormycosis has been documented. While this opportunistic infection generally affects immunocompromised individuals or patients with uncontrolled diabetes, mucormycosis can also present following traumatic injuries. Infection following motor vehicle accidents accounts for as little as 3% of traumatic cutaneous mucormycosis cases, however, it can have devastating consequences. In this report, we present a case of a 54-year-old male who acquired cutaneous mucormycosis following a motorcycle accident. The patient was treated for multiple traumatic orthopedic injuries and remained intubated in the ICU for several days due to his critical condition. Shortly after extubation on hospital day five, lower extremity ischemia and necrosis began to develop as a result of poor tissue perfusion. Extensive serial debridements were performed and empiric IV antibiotics were initiated for presumptive bacterial necrotizing soft tissue infection. Necrosis continued to rapidly progress despite intervention, and eventually, care was withdrawn. We provide a discussion of this case to highlight the importance of including rare fungal infections in the differential diagnosis early in the clinical course to mitigate fatal complications.

## Introduction

Mucormycosis is a rare fungal infection characterized by rapidly progressing infarction and necrosis of host tissue, frequently resulting in death. It is most well-known for causing a devastating rhino-orbital-cerebral infection in humans; however, pulmonary, cutaneous, gastrointestinal, and renal infections are also possible. The infection is seen almost exclusively in immunocompromised individuals or patients with diabetes mellitus, likely because of the organism’s propensity to thrive in high glucose, acidic conditions such as the serum of individuals in diabetic ketoacidosis [1]. The exact number of cases of mucormycosis is difficult to determine because no national surveillance exists in the United States, however, one study from the San Francisco Bay area found the annual incidence to be 1.7 cases per one million individuals from 1992 to 1993 [2]. A larger, population-based study in France found 0.7 cases per one million in 1997, rising to 1.2 per million in 2006 [3]. Despite its low incidence, mucormycosis is generally lethal, with an overall mortality of 54% among the total population and 96% in disseminated disease [4]. The genera most commonly found in human infections are *Rhizopus*, *Mucor*, and *Rhizomucor*, all of which are easily identifiable in clinical specimens as broad, irregularly branched hyphae with rare septations [5]. The rapidly progressing necrosis seen in mucormycosis is a result of these hyphae invading the vasculature, with subsequent thrombosis and vessel occlusion [6]. The most common clinical presentation is a rhino-orbital-cerebral infection consisting of fever, sinusitis, headache, and nasal ulceration/discharge which quickly progresses to a necrotic black eschar visible in the nasal mucosa, palate, or orbit [4,7]. Cutaneous mucormycosis is a less common variation resulting from the inoculation of fungal spores from dirt/soil into the dermis following trauma [8]. Compared to other forms of mucormycosis, cutaneous mucormycosis has a better, yet still, unfavorable prognosis ranging from 26% to 43% mortality in deep tissue infections [9]. Penetrating trauma is the most commonly cited cause of traumatic cutaneous mucormycosis, followed by dressings, surgery, and burns in order of decreasing incidence. Motor vehicle accidents account for a minority of cases at only 3% [4]. In this report, we present a case of a 54-year-old male who acquired a cutaneous mucormycosis necrotizing soft tissue infection following a polytraumatic motorcycle crash.

## Case presentation

**Figure 1:**
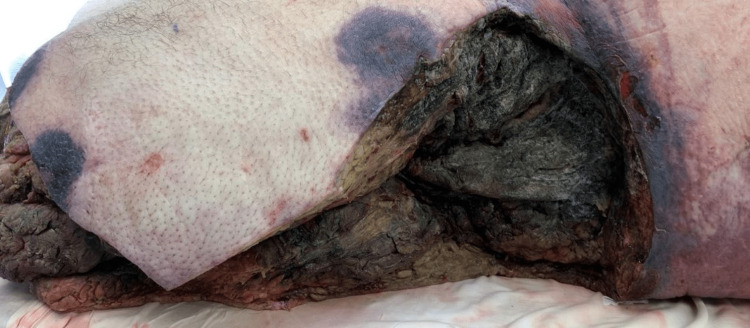
Left lateral thigh after AKA and multiple debridements with rapid progression of necrotic tissue AKA: above-knee amputation

A 54-year-old male with a history of type 2 diabetes mellitus was initially evaluated in the trauma bay after a high-speed collision with a motor vehicle as the helmeted driver of a motorcycle. The patient was reportedly found pinned underneath a car. On arrival, the patient’s primary survey was significant only for diminished right anterior tibialis pulse and absent left anterior tibialis pulse, with no abnormalities of airway or breathing and a Glasgow Coma Scale of 15. Preliminary X-ray imaging revealed a comminuted left intertrochanteric fracture, comminuted left proximal femoral fracture, open left tibia/fibula fracture, right proximal humerus fracture, and right distal ankle fracture, all later confirmed by computed tomography (CT). A secondary survey revealed the patient was covered in dirt with abrasions over the chest, abdomen, right shoulder and chest, bilateral elbows, and left lateral thigh. There were also open puncture wounds on the left shin and ankle. Additionally, a left patella degloving injury and obvious deformities of the bilateral lower extremities were identified, as well as discoloration of both feet. Further imaging revealed multiple bilateral rib fractures, a small left basilar pneumothorax, and a grade one splenic laceration. The patient was stabilized and remained intubated in the intensive care unit for the management of polytrauma as well as leukocytosis, acute blood loss anemia, lactic acidosis, thrombocytopenia, hyponatremia, hyperchloremia, hypocalcemia, and hypophosphatemia. Sliding scale insulin was used for the management of diabetes, with blood glucose levels usually falling between 120 and 220 mg/dL during the admission. Hemoglobin A1c was found to be 5.6%. The patient underwent several orthopedic surgeries over the course of the next two days for the management of his unstable lower extremity fractures. Rib fractures, pneumothorax, and splenic laceration were treated non-operatively. Vascular surgery was consulted two days after the initial trauma with concern for left foot ischemia considering swelling, coolness to touch, and absent anterior tibialis pulses on that side. CT angiography was done on hospital day two and revealed intermuscular edema of the lower extremities but was negative for arterial occlusion of the abdomen, pelvis, or lower extremities. Lack of obvious large vessel occlusion of the left lower extremity was confirmed by CT angiography the following day, however, vascular surgery determined that the forefoot was likely not viable based on a physical exam. The viability of the left lower extremity was monitored over the course of the next two days until ultimately the patient underwent a left below-knee amputation. Intraoperatively the left foot appeared dusky and bluish in color, with areas of black demarcation of necrosis visible at the distal ankle. After this procedure, the patient was extubated and was awake and alert with pain well controlled. Evaluation one day after extubation was significant for increasing mottling of the medial thigh with coolness to touch and mild tenderness; the patient was scheduled for medial thigh fasciotomy and incision and drainage the following day for suspected left thigh compartment syndrome with necrotic muscles. By surgery the following day, however, the patient’s necrotic wound had expanded, and the patient instead underwent a left above-knee amputation with left medial thigh fasciotomy. Approximately 24 hours later, the patient’s necrotic wound had now expanded to encompass the left knee amputation site, left flank, and left axilla. Working diagnosis at this time was septic shock and necrotizing fasciitis versus compartment syndrome versus both for which the patient underwent extensive excisional debridement of the affected areas. After consulting with Infectious Disease, the patient was started on intravenous vancomycin and Zosyn for coverage of commonly suspected crush injury organisms including *Clostridium perfringens*/*septicum*. Infectious disease at this time determined a very low suspicion for invasive fungal process considering the patient was not chronically immunocompromised, not a transplant or leukemia patient, and had good glycemic control of his diabetes with oral agents. Further extensive debridement of the same areas, as well as additional debridement of the right lower extremity and left hip disarticulation, were then performed with a continuation of empiric treatment with vancomycin and Zosyn. Vascular surgery was again consulted and deemed now the right lower leg to be non-viable and recommended a right above knee amputation. Several hours later, the patient was again taken to the operating room for this procedure as well as further debridement of the previously debrided areas due to the unrelenting progression of necrotizing soft tissue infection (Figure 1).

**Figure 2:**
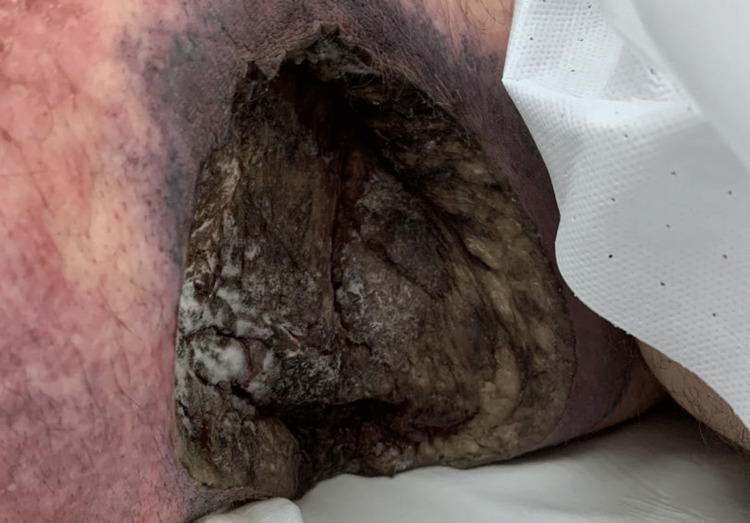
Left lateral chest wall with the apparent mold-like substance on the necrotic base

It was at this time that an initial examination of the wounds revealed a fuzzy, mold-like substance within the base of necrotic black and tan muscle (Figure 2).

**Figure 3:**
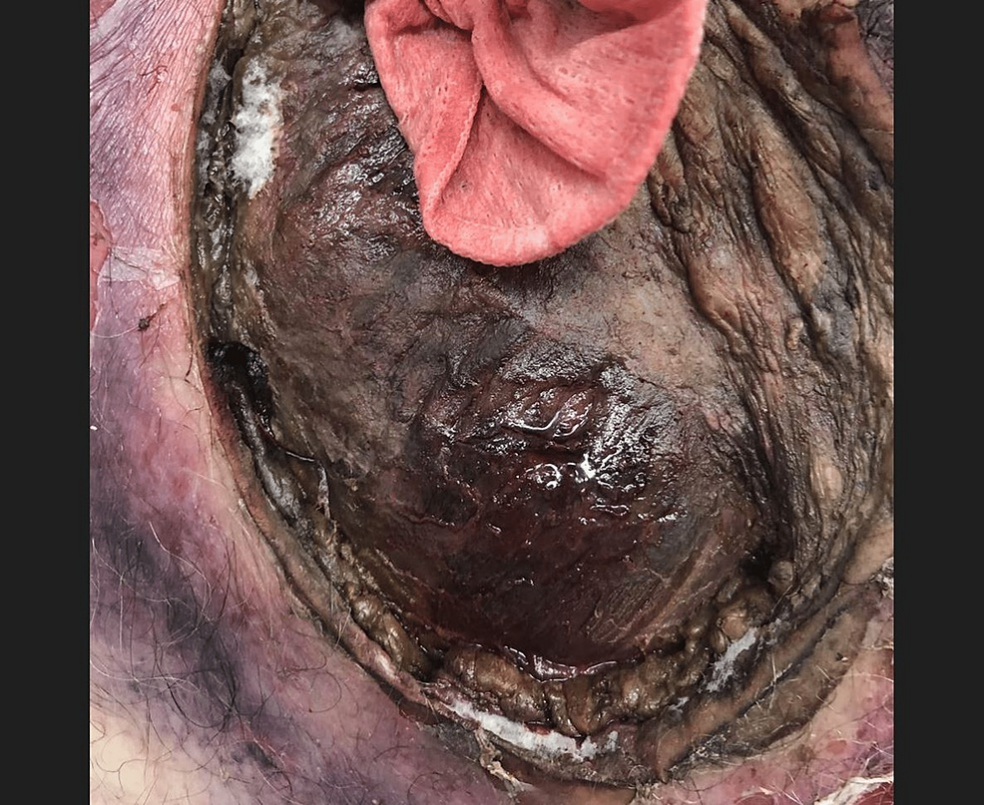
Left lateral chest wall approximately nine hours after prior debridement

Tissue assessed by the pathologist intraoperatively revealed a gross fungal infection. Another excisional debridement was performed approximately nine hours later with a re-demonstration of mold-covered necrotic tissue (Figure 3).

After mechanical debridement of the left thigh stump, abdomen, left flank, left lateral chest wall, and right posterior shoulder, each wound was packed with kerlix soaked in an amphotericin B/D5W mixture for treatment of suspected fungal infection. Approximately 12 hours after the completion of this final procedure, the patient’s family decided to withdraw care due to extensive morbidity. The patient was extubated on hospital day 12 following initial trauma and was pronounced dead shortly thereafter. Pathology results from specimens obtained during the last operation revealed fungal organisms morphologically consistent with mucormycosis.

## Discussion

Mucormycosis necrotizing soft tissue infection following a motor vehicle accident is a rare occurrence based on current literature, with one study reporting that motor vehicle accidents account for only 3% of traumatic causes of cutaneous mucormycosis [4]. Infection in such cases is likely a result of inoculation of a large fungal load directly into the dermis following a “road rash” type injury in an environment replete with dirt/soil colonized by a fungus of the *Mucorales *order. The patient presented in this case had just such an injury, evidenced by the multiple abrasions and significant amount of dirt covering the patient following the trauma. Vessel occlusion, a hallmark of the necrotizing infection caused by mucormycosis, was also evident in this case. Earliest signs of diminished extremity perfusion were on a primary survey in the trauma bay with diminished and/or absent anterior tibialis pulses and foot discoloration. Poor extremity perfusion continued to be evident on serial physical examinations, with a concern for left foot ischemia on hospital day three, although CT angiography was negative for large vessel occlusion. Further progression of ischemia resulted in a dusky, bluish discoloration of the extremities as well as mottling of the skin of the inner thigh, eventually leading to bilateral leg amputations and extensive debridement. Poor perfusion of the extremities seen early in the clinical course was likely a sequela of trauma and intermuscular edema, whereas ischemia observed towards the end of life was caused by mucormycosis invasion/occlusion of vessels. Unfortunately, the ischemia and eventual necrosis caused by mucormycosis were masked by ischemia resulting from significant trauma to the lower extremity. In other words, identifying that an infectious process was contributing to poor extremity perfusion was nearly impossible early on in the clinical course. Lastly, it should be noted that the timeline of this patient’s disease was not typical of a mold or fungal process. Mucormycosis is an opportunistic fungus with typically an acute onset and rapidly progressive nature [10]. Upon inoculation, necrosis is usually apparent within days. If we are to presume that this patient became infected during the motorcycle accident, then it appears that the fungus lied dormant for a number of days before beginning to show signs and symptoms typical of this disease. His history of diabetes is likely non-contributory considering his hemoglobin A1c of 5.6% and blood glucose levels which regularly remained between 120 and 220 mg/dL for the duration of his care. While this may not be ideal glucose control, it is certainly nowhere near values consistent with diabetic ketoacidosis needed to facilitate a mucormycosis infection. Then, perhaps the patient’s immune system was able to stave off cutaneous or disseminated infection early in the clinical course until his deteriorating condition from polytrauma temporarily compromised his immune system, allowing mucormycosis to flourish. The apparent indolent course observed in this case may provide some insight into the unique features of cutaneous mucormycosis infections associated with polytraumatic motor vehicle accidents.

## Conclusions

This case highlights the importance of including cutaneous mucormycosis in the differential of patients presenting with tissue ischemia following a road-rash type injury or dirty wounds in an area known to have endemic *Mucorales *species in soil. We encourage any clinician presented with a similar case to recognize large inoculation of fungal spores as an adequate risk factor for cutaneous mucormycosis infection, even in the absence of immunodeficiency, drastically elevated blood glucose, or other risk factors classically associated with mucormycosis. Early acknowledgment of potential fungal necrotizing soft tissue infection might result in earlier treatment which could prevent the fatal consequences of this rare, but extremely deadly saprophyte. Additionally, clinicians should be wary of a potentially more indolent course of such an infection in the setting of preceding trauma, a feature not typical of mucormycosis.
